# Genetic screening to avoid adverse drug reactions from medication use and approach patients' better outcomes: A lesson learn from the report of the Queen Savang Vadhana Memorial Hospital

**DOI:** 10.1002/hsr2.591

**Published:** 2022-04-13

**Authors:** Kessada Tunwongsa, Malinee Chonnawakul, Nopavut Geratikornsupuk, Karunrat Tewthanom

**Affiliations:** ^1^ Department of Pharmacy Queen Savang Vadhana Memorial Hospital Chonburi Thailand; ^2^ Department of Medicine Queen Savang Vadhana Memorial Hospital Chonburi Thailand; ^3^ Department of Pharmacy Silpakorn University Nakhon Pathum Thailand

**Keywords:** adverse drug reaction, genetics screening, lesson to learn, medication use

## Abstract

**Background and Aims:**

The polymerase chain reaction (PCR) technique is adopted for pharmacogenetic testing and adverse drug reaction (ADR) analysis. Methods: PCR was used for testing of pharmacogenetic markers for HLA and non‐HLA polymorphism related to specific drugs.

**Results:**

Among 76 cases that underwent genetic screening, 7.7%, 11.1%, and 2.7% of the patients were found to be genetically positive for allopurinol, carbamazepine, and abacavir, respectively. Two cases were genetically positive for interferon, and two cases of extensive metabolizers were positive for clopidogrel. One case of a NAT2 slow acetylator for isoniazid was found. Among the 74 cases with complete outcomes, 39.2% showed improvements and 18.9% reported a deterioration. Although no serious ADR was observed, two HLA‐B*5701‐negative cases reported ADRs (2.7%). All patients positive for IL28B were improved. One patient receiving clopidogrel showed improvements, but another showed deterioration. Finally, the outcome of slow acetylation NAT2 was worse without ADR.

**Conclusion:**

PCR‐based pharmacogenetic testing is critical for ADR monitoring in a cost‐effective manner.

## 1 INTRODUCTION

The relationships between genetics and adverse drug reactions (ADRs) and drug efficacy have been widely studied.^1^, ^2^, ^3^, ^4^, ^5^, ^6^, ^7^, ^8^, ^9^, ^10^ The literature has revealed the benefits of genetic screening.^4^ The Queen Savang Vadhana, a tertiary care hospital had patients receiving the drug that should be concerned about pharmacogenetics and ADR, therefore the Cooperation between the Department of Medical Sciences of the Ministry of Public Health and the Queen Savang Vadhana Memorial Hospital was established to develop better services for patient care. From the review literature, the National PGx guidelines, and Ministry of Public Health policies for considering the importance of PGx implementation for ADR prediction before selecting the appropriate drug for each patient,^11^ the major concern genes in ADR monitoring in the Thai population are HLA‐B*58:01 (frequency = 7.66%)^6^ for allopurinol, HLA‐B*15:02 (frequency = 6.38)^6^ for carbamazepine, and HLA‐B*5701 (frequency = 1.77)^6^ for abacavir. In addition, the evidence of the relationship between genetic and therapeutic efficacy in interferon,^5^, ^8^ clopidogrel,^9^ and isoniazid^10^ are available. Therefore, genetic screening for six drugs, including allopurinol, carbamazepine, abacavir, interferon, clopidogrel, and isoniazid, by using polymerase chain reaction (PCR) to determine the relationship between genetic factors and drug efficacy or ADRs^1^, ^2^, ^3^, ^4^, ^5^, ^6^, ^7^, ^8^, ^9^, ^10^ was implemented as a pilot project. The rational use of this technique is because of the available resources and facilities from the Department of Medical Sciences of the Ministry of Public Health. In addition, the benefits of genetic screening include improved therapeutic outcomes and lower incidence of ADRs due to genetic factors, including severe ADRs such as Stevens‐Johnson syndrome, toxic epidermal necrolysis, and drug reaction with eosinophilia and systemic symptoms syndrome.^12^ This pilot project aimed to report the results of the use of the PCR technique for genetic screening of various drugs, which could be used as a reference for future research.

## 2 METHODS

This project was approved by the internal ethics committee of the hospital (Project No. 005/2562) and the Ethics Committee of the Ministry of Public Health (Project No. IHRP2019054). The data were analyzed using descriptive statistics and presented as mean ± standard deviation and percentages.

### 2.1 Data collection

Data of genetic screening results were retrospectively collected. The information was retrieved from the hospital's computerized database from October 1, 2018, to October 31, 2020. The data were composed of demographic data from patients treated with six drugs, including allopurinol, carbamazepine, abacavir interferon, clopidogrel, or isoniazid, genetic screening results, and clinical outcomes.

### 2.2 Genetic screening procedure and analysis

The genetic screening procedure and analysis consisted of five steps as follows: DNA extraction, master mix solution preparation and DNA addition, PCR, agarose gel electrophoresis, and data interpretation.

Blood samples were collected into tubes with EDTA, and the DNA was extracted using an automatic DNA extractor (Zinext Life Science Corp.). This automated machine performs several steps to achieve DNA purification process, including lysis of the cell membrane, denaturation of proteins, DNA, and other macromolecules, promotion of nucleic acid binding to magnetic particles, removal of impurities, and collection of purified DNAs.

Next, the master mix solution was prepared and added with purified DNA. The mixed solution was composed of Taq polymerase, primers, and nucleotides (A, T, C, G), and buffer. The purified DNA was divided into three groups, including the samples (from patients), positive control (mutated‐type DNA), and negative control (wild‐type DNA). After mixing, all of the samples were transferred to PCR tubes.

The amount of DNA in the PCR tubes was increased by PCR in Bio‐Rad T100™ thermal cycler (Bio‐Rad Laboratories, Inc.). The PCR process comprised three steps: denaturation (strand separation 95°C), annealing (initiation of binding between the primer and the separated DNA at 55°C), and extension (synthesis of new DNA strands at 72°C).

All synthesized DNAs were analyzed by agarose gel electrophoresis with ethidium bromide. The data were interpreted as presented in Table 1.

**Table 1:** Demographic of patients who received genetic screening (*N* = 74)

Characteristics	No. cases (%)
Gender	
Male	52 (70.3)
Female	22 (29.7)
Age	47.77 ± 12. 77^a^
Diseases	
Gout	25 (33.8)
Epilepsy/neuropsychiatric	9 (12.2)
HIV infection	35 (47.3)
Chronic viral hepatitis	2 (2.7)
Heart disease	2 (2.7)
Tuberculosis	1 (1.3)
Drugs	
Allopurinol	25 (33.8)
Carbamazepine	9 (12.2)
Abacavir	35 (47.3)
Interferon	2 (2.7)
Clopidogrel	2 (2.7)
Isoniazid	1 (1.3)

Abbreviation: SD, standard deviation.

^a^ Mean ± SD.

Clinical outcomes were assessed based on standard clinical practice guidelines for each diseases^13^, ^14^, ^15^, ^16^, ^17^, ^18^ and classified as improved, unchanged, and worse. ADR evaluation was performed according to the Naranjo algorithm.^19^

## 3 RESULTS

### 3.1 Patient demographics

The demographic data of the patients are presented in Table 1. Most of the patients were male (70.3%), and the mean age was 47.77 years. Patients with HIV infection received abacavir was the highest rate, 35 (47.3%). Only one case of genetic screening for TB medication (isoniazid = 1.3%) was reported.

### 3.2 Genetic screening

The drugs, pharmacogenetic test, objective, and interpretation are demonstrated in Table 2. A summary of the number of patients and genetic screening results is presented in Table 3. A total of 76 cases were subjected to genetic screening. For allopurinol, only two cases of 26 were positive for HLA‐B*58:01 (7.7%). For carbamazepine, one in nine cases was positive for HLA‐B*15:02 (11.1%). Among 35 cases of abacavir, one case was positive for HLA‐B*5701 (2.7%). Two cases were positive for IL28B and equal to clopidogrel that had 2 cases of “extensive metabolizer.” Genetic screening for isoniazid revealed only one “slow acetylator.”

**Table 2:** The drugs, pharmacogenetic tests, objectives, and interpretation in genetic screening procedures

Drugs	Pharmacogenetics tests	Objective	Interpretation
1. Allopurinol	HLA‐B*58:01	ADR	Positive: increase risk of ADR
2. Carbamazepine	HLA‐B*15:02	ADR	Positive: increase risk of ADR
3. Abacavir	HLA‐B*5701	ADR	Positive: increase risk of ADR
4. Interferon	IL28B	Efficacy	Positive: increase risk of efficacy
5. Clopidogrel	CYP2C19	Efficacy	The different of activity of enzyme (extensive, poor metabolizer) are effect in efficacy
6. Isoniazid	NAT2	Efficacy	The different of activity of enzyme (slow, intermediate rapid) are effect in efficacy

Abbreviation: ADR, adverse drug reaction.

**Table 3:** Number of patients and genetic screening results

Drugs	Disease	Pharmacogenetics tests	Results	*N* (cases)	Total
1. Allopurinol	Gout	HLA‐B*58:01	Positive	2	26
			Negative	24	
2. Carbamazepine	Epilepsy/neuropsychiatric	HLA‐B*15:02	Positive	1	9
			Negative	8	
3. Abacavir	HIV infection	HLA‐B*5701	Positive	1	36
			Negative	35	
4. Interferon	Chronic viral hepatitis	IL28B	Positive	2	2
			Negative	0	
5. Clopidogrel	Heart disease	CYP2C19	Extensive metabolizer	2	2
6. Isoniazid (INH)	Tuberculosis	NAT2	Slow acetylator	1	1
			Intermediate	0	
			Fast acetylator	0	
Total					76

### 3.3 Genetic screening results and patients' outcomes

A total of 74 cases had completed information on their outcome assessment. Therefore, the data of these cases were used for analysis. These results are presented in Table 4. A few ADRs were identified. Two cases out of 35 (5.7%) had ADR from abacavir use. Only one patient who tested positive for genetic screening was prohibited from accepting the drug they reacted to. Therefore ADR did not occur. The percentage of patients with improved outcomes was higher than that of patients with poorer outcomes, although the percentage of unchanged outcomes was fairly high (64.7%) in patients receiving abacavir. The overall improvement was 39.2% (29 out of 74 cases), the overall deterioration was 17.6% (13 out of 74 cases), and the rest of the population reported no changes (43.2%; 39 out of 74 cases). One HLA‐B*58:01‐positive case revealed no ADR after receiving allopurinol. HLA‐B*58:01‐positive patients should generally be dissuaded from using allopurinol. Thus, this case was monitored for allopurinol treatment.

**Table 4:** Genetic screening results, ADR, and patients' outcome of drug treatment (*N* = 74)

Drug	Genetic results	No. case (%)	ADR	No. case (%)	Outcome	No. case (%)
1. Allopurinol (*N* = 25)	HLA‐B*58:01 positive	2 (8.0)	Absent	2 (100.0)	Improve	1 (50.0)
			Present	0 (0.0)	Worse	0 (0.0)
					Unchanged	1 (50.0)
	HLA‐B*58:01 negative	23 (92.0)	Absent	23 (100.0)	Improve	13 (56.6)
			Present	0 (0.0)	Worse	5 (21.7)
					Unchanged	5 (21.7)
2. Carbamazepine (*N* = 9)	HLA‐B*15:02 positive	1 (11.1)	Absent	1 (100.0)	Improve	1 (100.0)
			Present	0 (0.0)	Worse	0 (0.0)
	HLA‐B*15:02 negative	8 (88.9)	Absent	8 (100.0)	Improve	4 (50.0)
			Present	0 (0.0)	Worse	1 (12.5)
					Unchanged	3 (37.5)
3. Abacavir (*N* = 35)	HLA‐B*5701 positive	1 (2.9)	Absent	1 (100.0)	Improve	1 (100.0)
			Present	0 (0.0)	Worse	0 (0.0)
	HLA‐B*5701 negative	34 (97.1)	Absent	32 (94.1)	Improve	6 (17.6)
			Present	2 (5.9)	Worse	6 (17.6)
					Unchanged	22 (64.7)
4. Interferon (*N* = 2)	IL28B positive	2 (100)	Absent	2 (100.0)	Improve	2 (100.0)
			Present	0 (0.0)	Worse	0 (0.0)
	IL28B negative	0	Present	0 (0.0)	Improve	0 (0.0)
			Absent	0 (0.0)	Worse	0 (0.0)
5. Clopidogrel (*N* = 2)	CYP2C19 extensive metabolizer	2 (100)	Absent	2 (100.0)	Improve	1 (50.0)
			Present	0 (0.0)	Worse	1 (50.0)
6. Isoniazid (INH) (*N* = 1)	NAT2 slow acetylator	1 (100)	Absent	1 (100.0)	Improve	0 (0.0)
			Present	0 (0.0)	Worse	1 (100.0)

Abbreviation: ADR, adverse drug reaction.

## 4 DISCUSSION

The cooperation with the Department of Medical Sciences of the Ministry of Public Health to improve patients' care services by using PCR technique in genetic screening to avoid ADR of six medications found that the prevalence of HLA‐B*58:01‐positive cases in this study is similar to that determined in a previous report (7.4% vs. 7.7%).^13^ None of the ADR cases was severe, and the affected patients were managed or provided with alternative treatments. Screening of non‐HLA related genetics for ADR; NAT2 slow acetylator for isoniazid, found that high frequency of NAT2 slow acetylator in this study (1/1 = 100%). NAT2 enzyme activity is the most widely studied association with a non‐HLA gene to date for drug‐induced liver injury. Similarly in Malaysia, more than half of the studied population (64%) was NAT2 slow acetylator.^19^, ^20^ Although the single NAT2 slow acetylator case did not report an ADR, the outcome of the patient was poorer. Thus, whether the findings are related to NAT2 activity should be clarified in future research. Although no conclusions regarding the relationship between NAT2 activity and DLI were obtained, this study detected a problem during treatment with isoniazid in a NAT2 slow acetylator patient. A small randomized controlled trial suggested that the NAT2 genotype‐guided regimen could reduce isoniazid‐induced liver injury and early treatment failure in the 6‐month four‐drug standard treatment of tuberculosis.^21^ A recent pharmacokinetic cohort study found that isoniazid concentration and NAT2 genotype can predict the risk of systemic drug reactions during tuberculosis treatment.^22^

The results of this study on the outcomes of interferon and clopidogrel are similar to those of previous researchers. Among two patients receiving clopidogrel, one (50%) showed improvements but the other revealed deterioration. It exhibited a variety of responses by using genotyping as a comment on the previous research.^9^ Several factors, including noncompliance, drug‐drug interactions, and relevant comorbidities, may influence the results. Therefore, whether routine genetic screening for clopidogrel is recommended for all patients may be debated. The results of interferon are also in line with a previous study conducted in Poland on the association of IL28B polymorphisms with the response to peginterferon plus ribavirin combined therapy.^8^ In the present study, two IL28B‐positive patients showed improved outcomes. No IL28B‐negative patient was found in this study.

Genetic screening via the PCR technique before the administration of allopurinol, carbamazepine, abacavir, interferon, clopidogrel, and isoniazid revealed low ADR rates, and approximately 40% of the patients showed some improvement. Some studies have indicated that genetic screening of HLA‐B*5801 for allopurinol in the Asian and African American populations may be cost effective^11^ because doing so could reduce the expenditure for ADR management on account of the high percentage of HLA‐B*5801 of allopurinol in these populations. Therefore, the implementation of genetic screening for HLA‐B*5801 in these populations may be considered.

The PCR technique is capable of rapid results and has a reasonable cost. As cases of ADR increase, expenditures may be expected to decrease because the price of materials could be negotiated; however, cost‐effectiveness studies are still necessary to assess this inference. A study on the cost‐effectiveness of genetic screening in Thailand is ongoing.

The limitation of this report is the insufficient sample size for a general conclusion. In addition, it is not only one candidate gene that is responsible in ADR, therefore, A set of biomarkers (more than one marker) may be applied in this population, especially in negative results from the first biomarker. Further, studies are needed to identify more candidate genes that may have a relationship to ADR.

## 5 CONCLUSION

The PCR technique could be used for the genetic screening of patients with specific genetic polymorphisms related to drug toxicity (e.g., allopurinol, carbamazepine, abacavir) or drug effectiveness (e.g., interferon, clopidogrel, isoniazid) to minimize ADRs and enhance the efficacy of drug therapy. Implementation of this technique in routine settings requires further evaluation via cost‐effectiveness studies.

## AUTHOR CONTRIBUTIONS

*Conceptualized* **:** Karunrat Tewthanom, Kessada Tunwongsa. *Data curation*: Kessada Tunwongsa, Malinee Chonnawakul, Karunrat Tewthanom. *Investigation*: Nopavut Geratikornsupak. *Formal analysis* **:** Karunrat Tewthanom, Kessada Tunwongsa. *Writing and manuscript preparations and submission* **:** Karunrat Tewthanom. *Reviewing and Editing manuscript* **:** Kessada Tunwongsa, Malinee Chonnawakul, Nopavut, Geratikornsupak.

## CONFLICTS OF INTEREST

The authors declare no conflicts of interest.

## TRANSPARENCY STATEMENT

We confirm that this manuscript is an honest, accurate, and transparent account of the study being reported; that no important aspects of the study have been omitted; and that any discrepancies from the study as planned (and, if relevant, registered) have been explained.

## Data Availability

The authors confirm that the data supporting the findings of this study are available within the article.
